# Reduced sB7-H3 Expression in the Peripheral Blood of Systemic Lupus Erythematosus Patients

**DOI:** 10.1155/2017/5728512

**Published:** 2017-12-20

**Authors:** Jing Sun, Huijun Lai, Dong Shen, Pingping Wu, Jie Yang, Zhongwen Sun, Yundi Guo

**Affiliations:** ^1^Institute of Medical Biotechnology, Suzhou Vocational Health College, Suzhou, Jiangsu 215009, China; ^2^Ultrasound Institute, Suzhou Hospital of Traditional Chinese Medicine, Suzhou, Jiangsu 215009, China; ^3^Department of Clinical Laboratory, Suzhou Science and Technology City Hospital, Suzhou, Jiangsu 215009, China

## Abstract

Both membrane-bound and soluble forms of costimulatory molecules play important roles in immune-regulatory networks. B7-H3, a member of the B7 family, has been found with aberrant expression in tumors and infectious disease. However, the significance of sB7-H3 expression in systemic lupus erythematosus (SLE) has not been investigated. Using the peripheral blood of 78 SLE patients, we established a comprehensive database containing clinical data and relevant laboratory tests. We found that sB7-H3 expression in SLE patients was significantly lower compared with the healthy individuals. In addition, sB7-H3 levels in the patients were positively correlated with the disease activity as indicated by SLE disease activity index score, rashes, fever, and inflammatory cytokines. Moreover, sB7-H3 was associated with the counts of red blood cells and hemoglobin. Our findings suggest that sB7-H3 might counteract the aberrant immune response and potentially serve as a monitoring indicator of disease progression and therapeutic target in SLE treatment.

## 1. Introduction

Systemic lupus erythematosus (SLE) is a prototypic autoimmune disease characterized by circulating autoantibodies and the formation of immune complexes. Its main manifestations include hypergammaglobulinemia, immune complex formation, and activation of complement system. SLE autoantibodies have a highly specific reaction against autoantigens [1]. Furthermore, abnormal activation of immune cells in SLE patients occurs in T cells and antigen-presenting cells (APC), leading to immune disorders [2].

Signal transduction between immunocompetent cells is accomplished by the activation of intracellular costimulatory molecules. There are two major signaling pathways engaged in T cell activation: one is the response of T cell receptors (TCRs) to an antigen; the other is the stimulation of costimulatory molecules and their receptors. The second signal (signal 2) is provided by the interaction between APC and T cell costimulatory molecules. The positive costimulatory molecules (e.g., CD28, ICOS, ICAM-1, and LFA-3) and the negative costimulatory molecules (e.g., CTLA4, BTLA, and PD-1) form a complex signaling network to comprehensively regulate the immune reaction and play key roles in maintaining appropriate balance of immune activation and tolerance [3, 4].

B7-H3, a member of the B7 costimulatory molecule family, was initially identified in 2000 [5]. Since its discovery, investigators have focused on its biological functions in tumor immunity [6–8]. Furthermore, many studies also demonstrated that the soluble form of B7-H3 (sB7-H3) was aberrant in malignant tumors and correlated with the poor prognosis, and sB7-H3 could be the potential diagnostic and therapeutic target in tumor diseases [9–11]. There are few reports about the correlation between the B7-H3 molecule and autoimmune disease. Until now, few clinical reports demonstrated the role of B7-H3 in autoimmune diseases besides previous studies found altered soluble B7-H3 expression in rheumatoid arthritis and multiple sclerosis disease and correlation with clinical parameters [12, 13]. In the present study, we aimed to evaluate the expression of soluble B7-H3 in the SLE patients and determine whether its expression levels are related to the SLE disease state. These studies could interpret the mechanism of B7-H3 in autoimmune disease and assess if B7-H3 could be the therapeutic target in SLE. We collected the peripheral blood of 78 SLE patients and employed ELISA technique to identify the soluble B7-H3 (sB7-H3) expression pattern and further evaluated its correlation with the degree of disease activity, clinical manifestations, laboratory test indicators, and SLE-related inflammatory cytokine levels.

## 2. Subjects and Methods

### 2.1. Subjects

This study included 78 SLE patients with their peripheral blood collected in the Department of Rheumatology, Suzhou Hospital of Traditional Chinese Medicine, Jiangsu Province, China, from January 2013 to July 2015. All patients fulfilled at least four SLE diagnostic criteria published by the American College of Rheumatology. Among those 78 patients, 14 patients of which were newly diagnosed and 9 patients had never received any treatment prior to the blood draw. The other 55 patients received immunosuppressive therapy or hormonal therapy. The disease activity score of SLE was evaluated by the systemic lupus erythematosus disease activity index (SLEDAI) score. We divided the SLE patients into two groups (active and inactive phases) based on the degree of disease activity as assessed by the scores of SLE disease activity index (SLEDAI). Patients with SLEDAI scores of ≥6 were classified to be in the active phase. Those with 0–5 SLEDAI scores were classified to be in the inactive phase. The research protocol of this study was approved by the Ethics Committee of Suzhou Vocational Health College. Among the 78 SLE patients, 75 subjects were females, with an average age of 38.7 ± 15.0 years and the average course of the disease of 1.46 ± 2.10 years. Peripheral blood of 52 healthy volunteers who had normal physical examinations was collected in May 2015 as control subjects. These healthy volunteers had no history of allergy and autoimmune disease. There were no significant differences of age between the SLE and the healthy control groups (Table 1). The peripheral blood was collected and a centrifugation was followed to obtain serum. Collected serum was stored at −80°C for later analysis.

### 2.2. sB7-H3 Measurement

Serum levels of sB7-H3 were determined by ELISA using a kit obtained from eBioscience (San Diego, CA). Thawed serum samples were plated in triplicate into 96-well microplates, and ELISA was conducted following the manufacturer's instructions. Plates were read in a microplate reader (Bio-Rad, Hercules, CA) for the absorbance at 450 nm.

### 2.3. Serum Ig, C-Reactive Protein (CRP), Complement, Erythrocyte Sedimentation Rate (ESR), Standard Urine, and Blood Measurement

The concentrations of serum immunoglobulin G (IgG), CRP, C3, and C4 were determined by nephelometry methods according to the instructions described by the manufacturer (IMMUNE 800, Beckman Coulter). ESR and standard urine and blood tests were conducted according to the manufacturers' instructions.

### 2.4. Serum Cytokines

Serum cytokines interleukin (IL)-6, interferon gamma (IFN-*γ*), tumor necrotic factor-alpha (TNF-*α*), IL-4, and IL-17 were quantified using LEGENDplex™ Multiplex cytokine bead-based assay (BioLegend, San Diego, CA) and according to the manufacturer's instruction. For each reaction, 25 *μ*l of serum was diluted with 125 *μ*l deionized water prior to the test. All samples were run on an Accuri C6 flow cytometer (BD Biosciences, San Diego, CA) and acquired data were analyzed using LEGENDplex v7.0 software.

### 2.5. Statistical Analysis

Statistical analysis was conducted using GraphPad Prism 5 software (GraphPad software Inc., La Jolla, CA). The *t-*test was used when normal data distribution was confirmed; otherwise, the nonparametric Mann–Whitney *U* test was used to analyze the data. Nonlinear regression analysis was used to determine the correlation between the clinical examination indices and degree of disease activities as well as the correlation between sB7-H3 expression and inflammatory cytokine levels (IL-6, IFN-*γ*, TNF-*α*, IL-4, and IL-17). The correlations were determined based on the *r*^2^ values. *P* < 0.05 was considered statistically significant.

## 3. Results

### 3.1. SLE Patients Had Lower Serum Levels of sB7-H3

**Figure 1:**
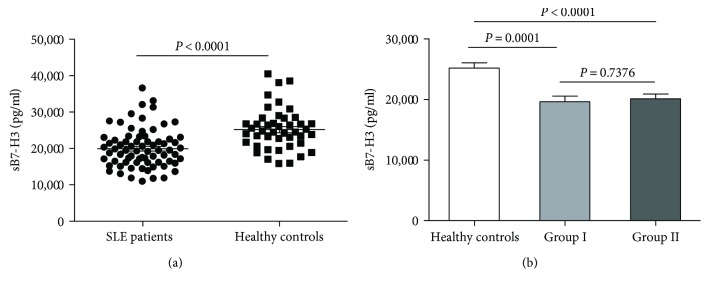
Serum levels of sB7-H3 in healthy control subjects and SLE patients. (a) SLE patients (*n* = 78) had lower serum levels of sB7-H3 compared to healthy control subjects (*n* = 56). (b) Serum levels of sB7-H3 were similar in group I which never underwent treatment group and group II which underwent treatment three months ago.

Results of ELISA showed that serum sB7-H3 concentrations in the SLE patients were significantly lower than those in the healthy individuals (19,930± 629.4 pg/ml versus 25,170 ± 857.7 pg/ml, *P* < 0.0001) (Figure 1(a)). Is the decrease of sB7-H3 in SLE patients because of using the hormonal drugs before? In order to exclude the effect of hormonal therapy on sB7-H3 expression, we divided the SLE patients into two groups; group I, 23 cases, which never undergone treatment group including newly diagnosed patients and group II, 55 cases, which undergone treatment before. The serum sB7-H3 levels in group I and group II were 19,620+ 925.0 pg/ml and 20,080+ 821.8 pg/ml, respectively, both of which were significantly lower than the serum levels of sB7-H3 in the healthy individuals (*P* = 0.0001 and *P* < 0.0001, resp.); however, no significant difference in sB7-H3 expression was found between these two groups (Figure 1(b)), suggesting that previous hormonal treatment had no impact on the sB7-H3 expression.

### 3.2. sB7-H3 Expression Correlated with the Degree of Disease Activities

**Figure 2:**
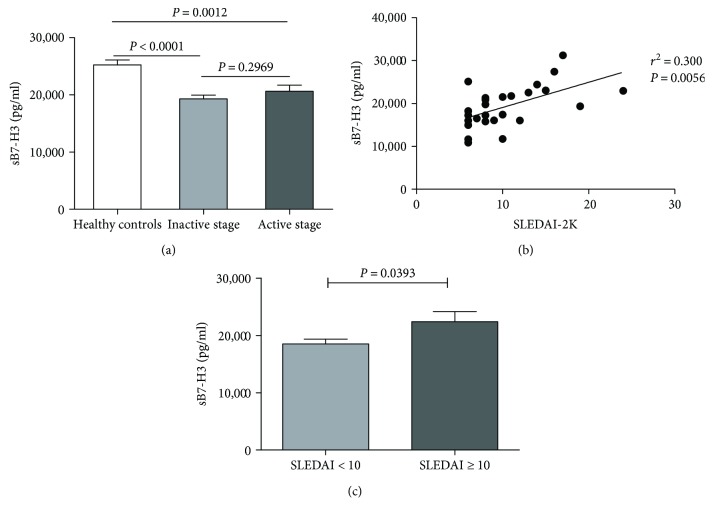
Correlation between serum levels of sB7-H3 and disease activity of SLE patients. (a) Similarly, lower serum levels of sB7-H3 were observed in the SLE patients in both active and inactive stages compared to the healthy control subjects. (b) Serum levels of sB7-H3 in SLE patients correlate with their disease activity score (SLEDIA) in active stages (*P* = 0.0056). (c) Significant lower serum levels of sB7-H3 in SLE patients with SLEDIA score less than 10 compared to patients more than 10.

In this study, approximately 53% of all SLE patients were in the active phase group with SLEDAI scores of ≥6. Serum sB7-H3 expression in the SLE patients who were in either active (20,590+ 1082 pg/ml) or inactive phase (19,460+ 641.7 pg/m) was significantly lower than that in the healthy individuals. Serum levels of sB7-H3 in the SLE patients of the active phase group were slightly higher than those in the SLE patients of the inactive phase group (Figure 2(a)), while these patients with SLEDAI scores exceeding 10 had significant higher sB7-H3 levels than these patients less than 10 (Figure 2(c)). To determine the relationship between sB7-H3 and the disease activity, we conducted correlation analysis and found a positive correlation (*P* = 0.0056) between sB7-H3 expression and SLEDAI scores, that is, SLE patients with higher sB7-H3 expression levels had higher SLEDAI scores (Figure 2(c)). To further determine whether sB7-H3 was related to the disease process of SLE, we analyzed the sB7-H3 expression and different clinical manifestations of SLE. The results showed that sB7-H3 expression in the SLE patients with cutaneous manifestations, Raynaud's phenomenon, and manifestations of the circulatory system (e.g., erythrocytopenia, thrombocytopenia, and anemia) was significantly higher than in SLE patients without the above manifestations (Table 2). Despite proteinuria, sB7-H3 expression in the SLE patients with other clinical manifestations was numerically higher than in the SLE patients without the clinical manifestations, although the difference was not statistically significant.

### 3.3. Correlation between sB7-H3 Expression and Clinical Laboratory Indicators

**Figure 3:**
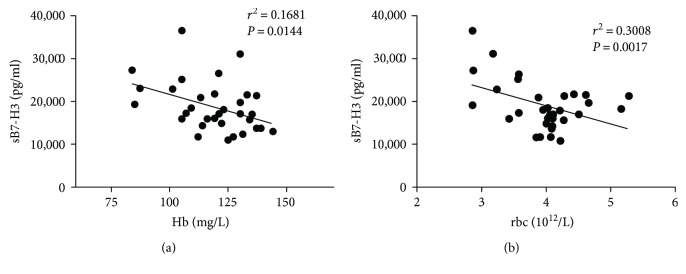
Negative correlation between serum levels of sB7-H3 and SLE laboratory tests in the active SLE patients. Serum levels of sB7-H3 were inversely correlated with Hb concentrations (a) (*P* = 0.001) and red blood cell counts (b) (*P* = 0.0091).

SLE patients often exhibit abnormal blood profile including leukopenia, thrombocytopenia, decreased hemoglobin, and reduced number of lymphocytes. Therefore, changes in these clinical laboratory indicators could be used as the disease activity markers of SLE. In this study, we performed correlation analysis between the sB7-H3 expression of SLE patients and the aforementioned clinical laboratory indicators (Table 3). Results showed that sB7-H3 expression of SLE patients was inversely correlated with the concentrations of hemoglobin and red blood cell counts in the peripheral blood (Figure 3).

### 3.4. Correlation between sB7-H3 Expression and Serum Cytokine Levels in the SLE Patients

**Figure 4:**
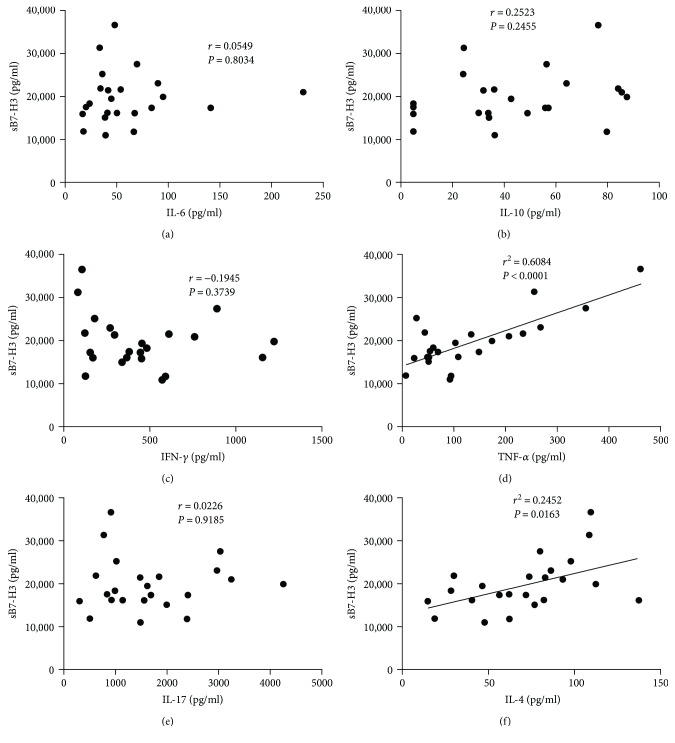
Correlation between serum levels of sB7-H3 and cytokines in the active SLE patients. Serum levels of sB7-H3 were positively correlated with TNF-*α* (d) (*P* < 0.001) and red blood cell counts (f) (*P* = 0.0163).

SLE patients have been shown to have abnormal activation of B cells and increased T cell differentiation into Th2, which facilitates differentiation of B cells into plasma cells to produce a large amount of antibodies. To evaluate whether B7-H3 plays a role in the process of T cell differentiation, we measured serum levels of cytokines (TNF-*α*, IFN-*γ*, IL-4, IL-6, and IL-10) which to a certain degree reflect a relative abundance of subset helper T cells. We found no significant correlation between sB7-H3 expression of SLE patients in inactive phase and serum levels of cytokines mentioned above. However, sB7-H3 expression of SLE patients in active phase was positively correlated with serum levels of TNF-*α* (*P* < 0.0001) and IL-4 (*P* = 0.0163) (Figure 4).

## 4. Discussion

Costimulatory molecules play an important role in the regulation of the immune response in autoimmune diseases, and therefore they may be useful as early diagnosis and treatment indicators of the disease. The most well-studied examples for this clinical function are PD-L1 and CTLA-4 [14]. A variety of costimulatory molecules exists both in membrane-bound and soluble forms, such as CD28, CTLA-4, CD80, CD86, ICOSL, B7-H3, and B7-H4. Soluble costimulatory molecules could be generated from proteolytic cleavage, such as ICOS and PD-L1 [15], and/or generated from mRNA splicing as PD-1 and CTLA-4 [16, 17]. Soluble costimulatory molecules play an important role in the immune-regulatory network [18]. Some soluble costimulatory molecules, such as sCTLA-4, can interfere the binding between CD80/CD86 and membranous CTLA-4 to block the inhibitory effect of CTLA-4, thus help activate T cells and enhance the immune response [19]. Consistent with this, highly expressed sCTLA-4 is found in a variety of autoimmune diseases. However, some soluble costimulatory molecules are functionally similar to membrane-bound CTLA-4, triggering the corresponding ligand or receptor such as soluble ICOS that activates ligand B7-H2, thereby enhancing the immune response [20].

Although its mRNA expression is varied, B7-H3 protein expression is limited and is known to function only in activated lymphocytes and tumor cells [21, 22]. Previous research suggests B7-H3 had inhibitory function in immune regulation [23, 24]; however, recent papers suggest that B7-H3 enhances T cell proliferation and cytotoxicity, as well as IFN-*γ*, TNF-*α*, and IL-10 production [5]. Furthermore, stimulation of the receptor expressed on a myeloid cell- (TREM-) like Transcript (TLT-2) molecule on T cells promotes binding to B7-H3 and enhances T cell effector functions, such as proliferation, cytokine production, and cytotoxicity. In our previous study, we found elevated B7-H3 expression provides an indicator of a more severe active status of RA and could potentially be involved in the progression of diseases through informatory cytokine secretion. However, to the best of our knowledge, no information is available as far as SLE is concerned. sB7-H3 is generated due to the cleavage of mB7-H3 by metalloproteinase 2 (MMP2) [25]. Although an abnormal sB7-H3 expression has been reported in patients with cancer and bacterial infection, the exact biological mechanism remains unclear [26, 27].

In this study, we showed that sB7-H3 expression in SLE patients was lower than in healthy individuals. Reduction of sB7-H3 expression in SLE patients was consistent with the previous findings in other autoimmune diseases, such as rheumatoid arthritis and multiple sclerosis [12, 13]. In addition, sB7-H3 expression was positively correlated with the disease activity index and SLEDAI score in SLE. To elucidate that low sB7-H3 expression was a factor of previous hormonal therapy, we divided the SLE patients into subgroups based on whether the patients accepted therapy within three months before donating the blood. While sB7-H3 expression in either of the two groups of SLE patients was lower than in healthy individuals, no significant difference in sB7-H3 expression was found between the two subgroups of patients.

Low levels of a soluble molecule may be the consequence of two different processes, that is, low production or increased depletion. The first process, low production, was refuted for the reason that we found that the membrane B7-H3 was significantly upregulated on monocytes in SLE patients (data not shown). Therefore, it is possible that the low levels of sB7-H3 in SLE patients are the consequence of increased depletion in responding to the excessive immune response in the body. Potentially, free sB7-H3 in peripheral blood can bind to the relevant B7-H3 receptor on T cell surface to block the activation of mB7-H3 molecules that function in transferring positive signals and activating the immune response. No receptor for B7-H3 has yet been discovered, although there is some evidence that B7-H3 binds to the TLT-2 receptor on activated T cells as a costimulator [24, 28]. Some researchers are of the opinion that there are other possible receptors on activated T cells [23, 29]. Regardless of the identity of the B7-H3 receptor, B7-H3 can interact with some agent on the cell membrane to activate T cells. We speculate that soluble B7-H3 could bind the potential receptor by competition and could not play a costimulatory role in the immune response. The speculation was consistent with the study of the means by which soluble B7-H3 inhibits T cell activation in hepatocellular carcinoma [30]. Therefore, sB7-H3 might play a negative regulatory role in autoimmunity and suppress the activation of autoreactive T cells. Consequently, sB7-H3 binding to the cognate receptor in SLE patients led to lower sB7-H3 expression in the peripheral blood compared with the healthy population, because sB7-H3 is consumed during the neutralization of the activated-cognate receptors, the lower sB7-H3 presence in serum may indicate its effectiveness in blocking costimulation pathway to alleviate autoimmune response leading to the lower clinical activity of SLE patients. This speculation was confirmed by the results of correlation analysis of sB7-H3 expression in SLE patients with fever, anemia, and thrombocytopenia as we observed that sB7-H3 expression in those patients was higher than SLE patients without these clinical manifestations. As such, sB7-H3 plays a role in correcting the abnormally upregulated immune response in SLE and may potentially be a target molecule for SLE treatment.

Given the massive production of autoantibodies in SLE patients, clinical manifestations of this disease include leukopenia, reduction in proportion of red blood cells, thrombocytopenia, increased immunoglobulins, and decreased complement components. These manifestations could be used as disease activity indicators for SLE. Therefore, we performed correlation analysis between serum sB7-H3 expression in the SLE patients and these indices. Our results showed a significant negative correlation between serum sB7-H3 expression and the concentrations of hemoglobin and counts of red blood cells in SLE patients. These results suggested that sB7-H3 could be used as an evaluation indicator for disease activity of SLE.

Disturbance of T cell subset distribution and the activation and functional abnormality (including cytokine secretion) of different T cell subsets plays an important role in the pathogenesis of SLE. Abnormal activation of B cells causes T cells to differentiate into Th2 cells and promotes the differentiation of B cells into plasma cells to produce large amount of antibodies. To evaluate whether B7-H3 was important in T cell differentiation, we measured serum concentrations of cytokines TNF-*α*, IFN-*γ*, IL-4, IL-6, and IL-10 and found no significant correlation between sB7-H3 expression of SLE patients in inactive phase and the above serum cytokine profile. However, in a correlation analysis, sB7-H3 expression of SLE patients in active phase was positively associated with TNF-*α* and IL-4 levels. Therefore, we speculated that B7-H3 might induce autoreactive T cell differentiation into Th2 cells. Our findings were consistent with the study by Nagashima et al., which shows that B7-H3 promoted the pathogenic Th2 cell development in an asthma mouse model [31].

This is the first study to report reduced serum sB7-H3 expression in the peripheral blood of SLE patients, which positively correlated with disease activity. While the exact underlying mechanisms are yet to be further elucidated, one possible cause might be the immune response that attempts to correct the abnormal upregulation in autoimmune diseases. sB7-H3 may bind to the B7-H3 receptor to block the stimulatory signal of mB7-H3 and trigger a negative immunoregulatory mechanism. These findings suggest that sB7-H3 could be used to monitor disease progression of SLE patients and potentially serve as a therapeutic target in SLE treatment.

## Figures and Tables

**Table 1:** Demographic data of SLE patients and healthy control subjects.

	SLE patients (*n* = 78)		Healthy control (*n* = 52)	
	Range/female	Mean ± SD/male	Range/female	Mean ± SD/male
Age (years)	21–78	38.7 ± 15.0	19–72	35.7 ± 11.3
Gender	75	3	50	2
Age at onset (years)	19–77	42.3 ± 19.7	—	—
Duration of disease (months)	1–180	42.73 ± 43.11	—	—

**Table 2:** Correlation between sB7-H3 and clinical features.

Clinical features		Case	sB7-H3 (pg/ml)	*P*
			Mean ± SD	
Renal involvement	Yes	31	19,310 ± 858.1	0.8114
	No	47	18,900 ± 1236	
Arthralgia	Yes	36	19,550 ± 1395	0.5671
	No	42	18,610 ± 939.6	
Cutaneous manifestations	Yes	30	20,970 ± 1259	**0.0158**
	No	48	17,930 ± 1053	
Photosensitivity	Yes	12	19,110 ± 921	0.7383
	No	66	18,140 ± 994.1	
Alopecia	Yes	17	22,910 ± 733	0.1017
	No	61	18,290 ± 1165	
Oral ulcers	Yes	18	21,340 ± 2800	0.2391
	No	60	18,640 ± 855.4	
Fever	Yes	23	21,340 ± 2618	0.0968
	No	55	16,450 ± 1112	
Raynaud's phenomenon	Yes	16	24,110 ± 782	**0.0347**
	No	62	17,852 ± 1185	
Proteinuria	Yes	43	18,830 ± 1022	0.5552
	No	35	19,750 ± 1141	
Hypocomplementemia	Yes	54	19,350 ± 836.5	0.5178
	No	24	17,820 ± 1694	
Hyperimmunoglobulinemia	Yes	25	19,450 ± 1647	0.8827
	No	53	19,190 ± 927.2	
Erythrocytopenia	Yes	18	22,630 ± 2303	**0.0166**
	No	60	18,090 ± 719.8	
Leukopenia	Yes	26	20,820 ± 1860	0.1546
	No	52	18,360 ± 778.3	
Thrombocytopenia	Yes	11	23,450 ± 2740	**0.0217**
	No	67	18,060 ± 786.9	
Anemia	Yes	23	22,110 ± 1669	**0.0120**
	No	55	17,830 ± 797.2	

The *t*-test was used when the data distribution was confirmed as normal; otherwise, the nonparametric Mann–Whitney *U* test was used to analyze the data. *P* < 0.05 was considered as statistically significant. The numbers of SLE patients are 78.

**Table 3:** Correlation between laboratory tests and serum levels of sB7-H3 level in SLE patients.

Parameter	Range	Mean ± SD	*r* value	*P* value
WBC (10^9^/L)	3.2–12.88	5.84 ± 2.27	−0.1783	0.2253
RBC (10^12^/L)	2.56–5.28	3.97 ± 0.56	−0.3116	**0.0091**
Hb (mg/L)	76–152	118.61 ± 17.69	−0.3890	**0.0010**
PLT (10^9^/L)	47–349	207.18 ± 80.41	−0.1849	0.2083
IgG (g/L)	6.3–41.6	15.35 ± 7.01	0.01240	0.9371
IgA (g/L)	0.896–4.77	2.79 ± 1.08	0.1627	0.2973
IgM (g/L)	0.219–3.12	0.982 ± 0.57	0.06669	0.6709
IgE (g/L)	<17.1–111	235.6 ± 502	0.1076	0.4977
1st hour ESR (mm)	1–126	26.86 ± 28.8	0.04316	0.7861
C3 mg%	0.219–1.08	0.707 ± 0.202	−0.03821	0.8010
C4 mg%	<0.056–0.264	0.1429 ± 0.054	−0.0002820	0.9986
24 h urinary protein (g)	107–154	416.23 ± 462.8	0.1313	0.5503

WBC: white blood cell; RBC: red blood cell; Hb: hemoglobin; PLT: blood platelet; C3: complement 3; C4: complement 4; ESR: erythrocyte sedimentation rate. The numbers of SLE patients are 78. Nonlinear regression test was used to analyze the data. *P* < 0.05 was considered as statistically significant.
